# The Accuracy of Emergency Physicians in Ultrasonographic Screening of Acute Appendicitis; a Cross Sectional Study

**Published:** 2017-01-10

**Authors:** Ebrahim Karimi, Mohammad Aminianfar, Keivan Zarafshani, Arash Safaie

**Affiliations:** 1 Emergency Department, Be’sat Hospital, Aja University of Medical Sciences, Tehran, Iran.; 2 Department of Infectious and Tropical Diseases, Be’sat Hospital, Aja University of Medical Sciences, Tehran, Iran.; 3 Emergency Department, Sina Hospital, Tehran University of Medical Sciences, Tehran, Iran.

**Keywords:** Appendicitis, ultrasonography, decision support techniques, emergency service, hospital, diagnosis

## Abstract

**Introduction:**

Diagnostic values reported for ultrasonographic screening of acute appendicitis vary widely and are dependent on the operator’s skill, patient’s gender, weight, etc. The present study aimed to evaluate the effect of operator skill on the diagnostic accuracy of ultrasonography in detection of appendicitis by comparing the results of ultrasonography done by radiologists and emergency physicians.

**Methods:**

This prospective diagnostic accuracy was carried out on patients suspected to acute appendicitis presenting to EDs of 2 hospitals. After the initial clinical examinations, all the patients underwent ultrasonography for appendicitis by emergency physician and radiologist, respectively. The final diagnosis of appendicitis was based on either pathology report or 48-hour follow-up. Screening performance characteristics of appendix ultrasonography by emergency physician and radiologist were compared using STATA 11.0 software.

**Results:**

108 patients with the mean age of 23.91 ± 7.46 years were studied (61.1% male). Appendicitis was confirmed for 37 (34.26%) cases. Cohen's kappa coefficient between ultrasonography by the radiologist and emergency physician in diagnosis of acute appendicitis was 0.51 (95% CI: 0.35 – 0.76). Area under the ROC curve of ultrasonography in appendicitis diagnosis was 0.78 (95% CI: 0.69 – 0.86) for emergency physician and 0.88 (95% CI: 0.81 – 0.94) for radiologist (p = 0.052). Sensitivity and specificity of ultrasonography by radiologist and emergency physician in appendicitis diagnosis were 83.87% (95% CI: 67.32 – 93.23), 91.5% (95% CI: 81.89 – 96.52), 72.97% (95% CI: 55.61 – 85.63), and 83.10% (95% CI: 71.94 – 90.59), respectively.

**Conclusion:**

Findings of the present study showed that the diagnostic accuracy of ultrasonography carried out by radiologist (89%) is a little better compared to that of emergency physician (80%) in diagnosis of appendicitis, but none are excellent.

## Introduction

Appendicitis is a common surgical emergency in young adult males presenting to emergency department (ED) following abdominal pain (1, 2). Diagnosis of appendicitis is a challenge for the medical team, as most of the time its classic signs and symptoms, such as pain, are not present and laboratory tests do not have enough predictive value in this regard (3). Meanwhile, rapid diagnosis and timely treatment can improve these patients’ management and reduce their hospital stay (4). Laparotomy is the gold standard tool in diagnosis and treatment of appendicitis, but is invasive and has its own limitations and dangers. This has led the researchers to evaluate the diagnostic accuracy of other diagnostic tools such as computed tomography (CT) scan, magnetic resonance imaging (MRI), and ultrasonography in detection of appendicitis. Using each of these tests has its own advantages and limitations and the diagnostic values reported for them varies between different studies (5, 6). This might be the reason that unnecessary laparotomies still have a high prevalence in abdominal pains suspected to be appendicitis (3). Currently, bedside ultrasonography is deemed one of the most valuable screening tests in ED (-). This rapid diagnostic method can provide valuable results for the medical team in a short time with minimum cost (12). However, using ultrasonography for this purpose is still under debate. Diagnostic values reported for ultrasonography vary widely and are dependent on the operator’s skill, patient’s gender, weight, etc. (13). Yet, there is still no correct understanding of the mentioned factors on the diagnostic accuracy of ultrasonography. Therefore, the present study was done aiming to evaluate the effect of operator skill on the diagnostic accuracy of ultrasonography in detection of appendicitis by comparing the results of ultrasonography done by radiologists and emergency physicians.

## Methods

***Study design***

This prospective diagnostic accuracy was carried out on patients suspected to acute appendicitis presenting to EDs of Be’sat and Sina Hospitals, Tehran, Iran, during 2014 and 2015 with the aim of comparing the ultrasonographic screening results of patients by emergency physicians and radiologists. Protocol of the present study was approved by the Ethics Committee of Aja University of Medical Sciences. Before inclusion in the study, written informed consent for participation was obtained from all the patients or their relatives. The researchers adhered to the principles of the Declaration of Helsinki throughout the study.

***Participants***

Samples were chosen by probability convenience method. Patients with pain in the right lower quadrant of the abdomen suspected with acute appendicitis, who visited EDs of the 2 hospitals were included. Exclusion criteria consisted of acute abdomen in need of emergency laparotomy, confirmed diagnosis other than appendicitis (like ureteral stone), not undergoing ultrasonography by radiologist, emergency physician not being blinded to the result of the ultrasonography by radiologist, unavailability of laparotomy data or not being able to follow the patient, and discharge against medical advice. 

***Procedure***

**Figure 1:**
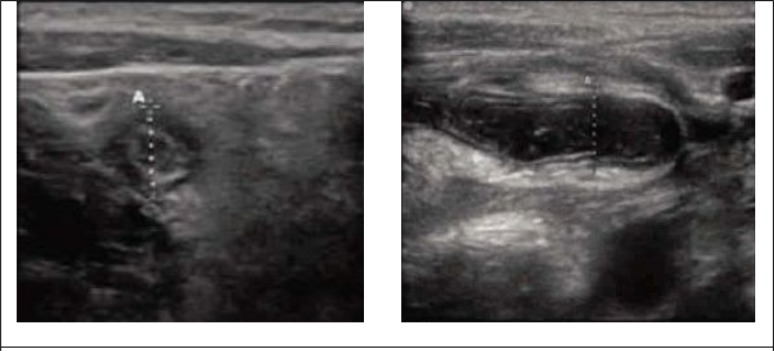
Sonographic views of appendix.

**Figure 2:**
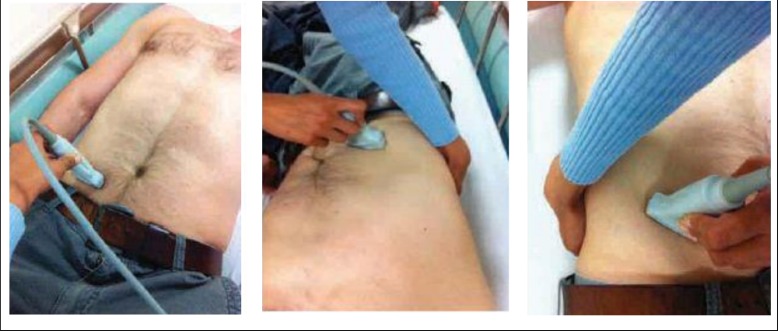
Location of probe for appendix ultrasonography.

After the initial clinical examinations, all the patients underwent ultrasonography for appendicitis by emergency physician and radiologist, respectively. The final diagnosis of appendicitis was based on pathology report (in patients undergoing surgery) or 48-hour follow-up (reference test). Ultrasonography was done using an ultrasonography machine (HS2000, Honda, Korea) with a linear probe and 5 – 7.5 MHz frequency. Ultrasonographic diagnosis of acute appendicitis (figure 1) was based on > 6mm outer diameter of the appendix, not being compressible, presence of appendicolitis, loss of bowel movements, and free fluid accumulation around the appendix (14, 15). The site of probe placement in the right lower quadrant was where the most tenderness was found in the clinical examination and to reduce bowel gases and the distance of the probe to appendix, a gentle continuous pressure was applied to the site before carrying out ultrasonography. Figure 2 shows the method of doing ultrasonography in patients. Then the patients were followed. If the patient was sent to the operation room, their pathology result was counted as the confirmed diagnosis of presence or absence of appendicitis. However, the final diagnosis of patients who did not undergo laparotomy and were only followed for at least 48 hours was determined based on their follow-up and with the help of other diagnostic tests such as CT scan. The diagnostic and treatment process of the patients was done without considering ultrasonography results. In addition, in patients who were discharged from ED, 48 hours after discharge, follow-up was done on the phone to evaluate the persistence or improvement of the symptoms and those who still had pain were invited to ED for further evaluation. These cases were followed and their final diagnosis was done based on laparotomy or further 48 hour follow up.

***Statistical analysis***

Minimum sample size needed for the present study was calculated to be 106 patients by considering 98.5% specificity of ultrasonography, 39.4% prevalence of appendicitis (12), α = 0.05 and d = 0.05. Data analysis was done using STATA 11.0 software. Area under the receiver operating characteristic (ROC) curve, sensitivity, specificity, positive and negative predictive value, positive and negative likelihood ratio and finally, Brier score of the emergency physician and radiologist for acute appendicitis diagnosis were compared. To evaluate the agreement between the results of the radiologist and emergency physician, Cohen's kappa coefficient was calculated. Presence of difference between the results of the emergency physician and radiologist was assessed using McNemar's chi square test. Diagnostic value of the test was considered excellent if between 90-100%, good if 80 - 90%, fair if 70 – 80%, poor if 60 – 70%, and fail if 50 – 60%. Significance level was considered p < 0.05.

## Results

108 patients with the mean age of 23.91 ± 7.46 years were studied (61.1% male). Finally, based on the reference test appendicitis was confirmed for 37 (34.26%) cases. Using ultrasonography, emergency physician and radiologist were able to diagnose 27 and 31 cases out of the 37, respectively. Cohen's kappa coefficient between ultrasonography by the radiologist and emergency physician in diagnosis of acute appendicitis was 0.51 (95% confidence interval (CI): 0.35 – 0.76). Area under the ROC curve of ultrasonography in appendicitis diagnosis was 0.78 (95% CI: 0.69 – 0.86) for emergency physician and 0.88 (95% CI: 0.81 – 0.94) for radiologist (figure 1). Although it seems that area under the curve for radiologist is higher than emergency physician, the difference is only borderline (p = 0.052). Area under the ROC curve of ultrasonography performed by radiologist in men (AUC = 0.86; 95% CI: 0.77 – 0.95) and women (AUC = 0.89; 95% CI: 0.77 – 1.0) was not different (p = 0.68). These rates were (AUC = 0.79; 95% CI: 0.69 – 0.89) for men and (AUC = 0.77; 95% CI: 0.61 – 0.92) for women in ultrasonography performed by emergency physician, which did not show a difference (p = 0.80) (figure 2).

Diagnostic value of ultrasonography by radiologist and emergency physician are reported in table 1. Sensitivity and specificity of ultrasonography by radiologist in appendicitis diagnosis were 83.87% and 91.5%, respectively. These values for emergency physician were 72.97% and 83.10%, respectively. The accuracies calculated were about 89% and 80%, respectively.

Brier score of the ultrasonography performed by radiologist was 0.11 and its scaled reliability was 0.01. These values were 0.20 and 0.05 for ultrasonography performed by emergency physician. These findings indicate the good predictive accuracy and reliability for both specialists in diagnosis of acute appendicitis using ultrasonography.

## Discussion

Findings of the present study showed that although diagnostic value of ultrasonography by radiologist is a little better than that of emergency physician, none are excellent. 

Reported diagnostic value of ultrasonography in appendicitis diagnosis varies between studies (16, 17). The results of a meta-analysis showed that sensitivity and specificity of ultrasonography in appendicitis diagnosis are 86% and 81%, respectively. Positive and negative predictive values reported in the study were 84% (46-95%) and 85% (60 – 97%), respectively. The researchers of the meta-analysis believe that a variety of factors are responsible for the difference between studies. The most important reported factor was the dependence of ultrasonography on operator’s skill (18). Although the findings of the present study confirms this hypothesis to some extent, the diagnostic value of ultrasonography by radiologist was not significantly different from that of emergency physician and the difference was on the borderline. 

In addition to the dependence of ultrasonography on the operator’s skill, some studies believed that the diagnostic value of ultrasonography is also dependent on the patient’s gender. These studies express that due to anatomic differences, differentiation of acute abdominal pains is very difficult in women of childbearing age (-). For this purpose, in the present study the diagnostic value of ultrasonography was evaluated based on patients’ gender. However, the findings showed that the diagnostic value of ultrasonography for appendicitis diagnosis does not vary between women and men.

Some studies have attempted to increase the sensitivity of ultrasonography by adding other diagnostic tests. For example Aspelund et al. added MRI and showed that in children suspected to appendicitis, a radiation-free diagnostic protocol screening the suspected cases, using ultrasonography and MRI, was a simple method and had a value equal to CT scan (22). In contrast, other studies have questioned the combined strategy for diagnosis of appendicitis. In a study, Leeuwenburgh et al. showed that although screening based on ultrasonography and CT scan for suspected patients has equal value to MRI, both methods classify about half of those with ruptured appendicitis as healthy. These researchers concluded that triage of appendicitis based on imaging is not appropriate for conservative treatment and may cause huge mistakes in patient management (23) since false positive results could lead to an increase in unnecessary appendectomy, while false negative results might cause a delay in treatment and therefore, worsening of the patient’s condition.

**Table 1:** Screening performance characteristics of ultrasonography performed by radiologist and emergency physician in detection of acute appendicitis

**Value**	**Radiologist**	**Emergency physician**
**True positive**	31	27
**True negative**	65	59
**False positive**	6	12
**False negative**	6	10
**Sensitivity**	83.78 (67.32-93.23)	72.97 (55.61-85.63)
**Specificity**	91.5 (81.89-96.52)	83.10 (71.94-90.59)
**Positive predictive value**	83.78 (67.32-93.23)	69.23 (52.27-82.45)
**Negative predictive value**	91.55 (81.89-96.52)	85.51 (74.49-92.46)
**Positive likelihood ratio**	9.91 (4.55-21-60)	4.32 (2.49-7-50)
**Negative likelihood ratio**	0.18 (0.08-0.37)	0.32 (0.19-0.56)
**Accuracy**	88.89 (82.96-94.48)	79.63 (72.03-87.23)

**Figure 3:**
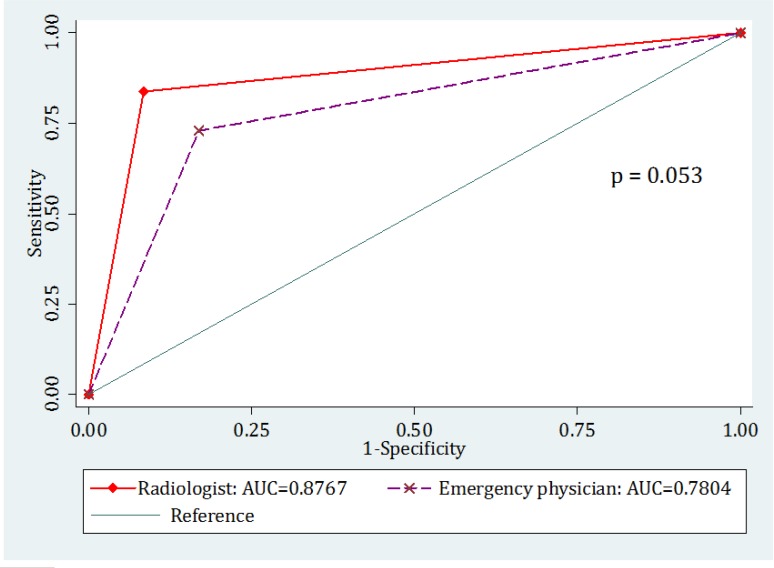
Comparison of area under the receiver operating characteristic (ROC) curve for ultrasonography carried out by radiologist and emergency physician

**Figure 4:**
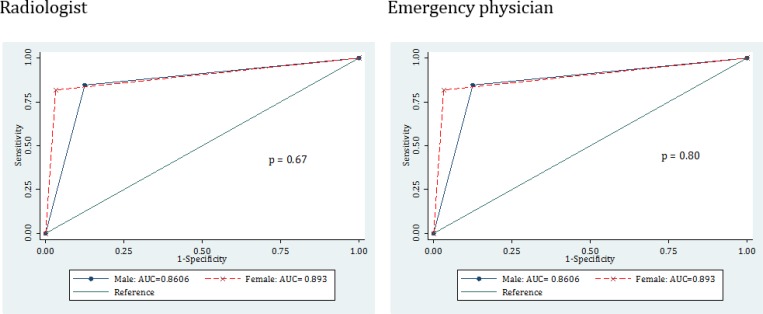
Comparing area under the receiver operating characteristic (ROC) curve of ultrasonography carried out by radiologist and emergency physician for diagnosing acute appendicitis based on patients’ gender

To solve this problem, the researchers suggest to not solely rely on imaging evaluations for diagnosis of appendicitis. Rather, use a mixture of diagnostic techniques including history taking and clinical examination, scoring systems, inflammatory biomarkers, in addition to imaging studies (18, 24). 

***Limitations***

Among the limitations of the present study is its observational nature .Therefore, eliminating all the confounding factors and probable biases from the study was not possible. Among these items are convenience sampling that makes selection bias probable. In addition, the present study is a 2-centered one and this makes generalizability of the data to all clinical conditions a bit hard. Finally, not evaluating weight and body mass index (BMI), which can affect the findings, is another limitation of this study.

## Conclusion:

Findings of the present study showed that the diagnostic accuracy of ultrasonography carried out by radiologist (89%) is a little better compared to that of emergency physician (80%) in diagnosis of appendicitis, but none are excellent. 
